# Fas Signaling Promotes Gastric Cancer Metastasis through STAT3-Dependent Upregulation of Fascin

**DOI:** 10.1371/journal.pone.0125132

**Published:** 2015-05-18

**Authors:** Yunshan Yang, Qiyu Zhao, Zhijian Cai, Guoping Cheng, Ming Chen, Jiaoli Wang, Haijun Zhong

**Affiliations:** 1 Department of Chemotherapy, Zhejiang Cancer Hospital, Hangzhou, 310022, People’s Republic of China; 2 Hepatobiliary & Pancreatic Intervention Center, Department of Hepatobiliary and Pancreatic Surgery, the First Affiliated Hospital, Zhejiang University School of Medicine, Hangzhou, 310058, People's Republic of China; 3 Institute of Immunology, Zhejiang University School of Medicine, Hangzhou, 310058, People’s Republic of China; 4 Department of Otolaryngology, the Second Affiliated Hospital of School of Medicine, Zhejiang University School of Medicine, Hangzhou, 310058, People’s Republic of China; 5 Department of Medical Oncology, Nanjing Medical University, Affiliated Hangzhou Hospital (Hangzhou First People’s Hospital), Hangzhou, 310006 People’s Republic of China; H.Lee Moffitt Cancer Center & Research Institute, UNITED STATES

## Abstract

**Background:**

Fas signaling-activated signal transducers and activators of transcription 3 (STAT3) is required for Fascin upregulation. As an actin-bundling protein, Fascin can mediate gastric cancer (GC) cell migration.

**Methods:**

Gastric cancer AGS cells were treated with anti-Fas (5 μg/ml) for 2 h, in order to stimulate the activation of the Fas signaling. The *in vitro* migration of Fas signaling-activated AGS cells was assessed using Transwell chambers. The levels of Fascin and phosphorylated STAT3 were detected by Western blotting analyses. Nude mice were injected intravenously with AGS cells treated with anti-Fas or treated with STAT3 inhibitor without anti-Fas; tumor pulmonary metastases were measured. Fascin protein expression in tumor tissues was detected by immunohistochemistry. The Fas and Fascin mRNA levels in tumor tissues from patients with GC were measured by real-time PCR and their correlation was analyzed.

**Results:**

The activation of Fas signaling promoted cell migration and resulted in STAT3-dependent Fascin upregulation in AGS cells. STAT3 enhanced Fascin levels *in vivo*. Fascin was the mediator of Fas signaling-induced AGS cell migration *in vitro* and *in vivo*. Furthermore, there was a positive correlation between Fas and Fascin mRNA levels in tumor tissues from GC patients.

**Conclusions:**

Fas signaling promotes GC metastasis through the STAT3/Fascin pathway, which may provide a new target for GC therapy.

## Introduction

Gastric cancer (GC) is the fourth most common cancer and the second leading cause of cancer-related deaths worldwide. Like most cancers, metastasis is the leading cause in failure of clinical therapy of GC. Diagnosed at an early stage without metastasis, GC can be eradicated by surgery. Once metastasis occurs, the prognosis of GC is significantly worse. In patients with extensive metastasis, the outcome of surgery combined with chemotherapy and immunotherapy is far from optimal, with an overall 5-year survival rate being only 24% [1,2]. Therefore, there is an urgent need for a better understanding of the mechanism of GC metastasis in order to develop a better therapeutic strategy.

Fas signaling pathway is one of the classical mechanisms to induce apoptosis [3]. After ligation with its natural ligand, FasL, Fas receptor forms the death-inducing signaling complex, causing the activation of caspase-8, which in turn activates the downstream caspases, resulting in apoptosis [4]. It has been reported that Fas-mediated cell killing is responsible for the anti-cancer function of Fas signaling pathway in prostate cancer [5]. However, in most tumors, instead of inducing apoptosis, Fas signaling activation promotes the tumor progression [6,7]. Tumor cells often respond to Fas stimulation with enhanced proliferation [8,9]. Several studies have also shown that Fas signaling can promote tumor cell migration and invasion [10–12]. In a recent study, high Fas expression in GC cells has been demonstrated to correlate with the occurrence of metastases to regional lymph nodes [13], suggesting that Fas signaling promotes the metastasis of gastric cancer.

Fascin, an actin-bundling protein, has been identified as a key molecule in tumor metastasis [14]. Fascin plays an important role in tumor cell migration and the expression of Fascin is increased in several types of cancers, including GC [15,16]. Fascin is a direct STAT3 target gene in response to IL-6 in both mouse and human breast cancer cells [17]. Fas signaling has been reported to be involved in the activation of STAT3 [18–20]. Therefore, we speculate that Fas signaling promotes the GC cell migration and subsequent tumor metastasis through the STAT3-dependent upregulation of Fascin.

The present study was designed to demonstrate the involvement of Fas in regulation of Fascin expression through STAT3 activation in AGS cells. We attempted to link the enhanced Fascin level with cell motility and metastasis of AGS cells *in vivo*. We also analyzed the correlation between Fas and Fascin mRNA levels in tumor tissues from patients with GC. It was hoped that our findings would reveal a novel mechanism for GC metastasis, providing a basis for the future development of Fas-based therapeutic strategy for advanced GC.

## Materials and Methods

### Human tissue specimens

Human GC tissue specimens were collected from 23 patients (14 with metastasis and 9 without metastasis) undergoing surgery before chemotherapy in Zhejiang Cancer Hospital (Hangzhou, China) between 2012 and 2014. Clinical characteristics of the patients with GC are summarized in Table 1. The study complied with the regulations of the Ministry of Health of China and the World Health Organization Research Ethics Review Committee international guidelines for research involving human subjects and the Declaration of Helsinki on the Ethical Principles for Medical Research Involving Human Subjects. The study protocol was reviewed and approved by the Institutional Review Board of Zhejiang Cancer Hospital. Written informed consent was obtained from each of the patients prior to study commencement.

**Table 1:** Patient characteristics.

Characteristics	Value
Number	23
Gender	
Male	13
Female	10
Age (years)	61.5 ± 4.1
ECOG PS(0–1)	23
Pathological grade(III)	23
*Clinical stage	
III	9
IV	14
Sites of metastases	
Liver	6
Ovarian	3
Peritoneum	5

NOTE: Data are mean ± standard deviation.

*According to American Joint Committee on Cancer.

Abbreviation: ECOG PS, Eastern Cooperative Oncology Group Performance Status.

### Reagents

Mouse anti-human Fas (2R2) monoclonal antibody was purchased from eBioscience (San Diego, CA, USA). Mouse anti-human Fas (CH11) monoclonal antibody and STAT3 inhibitor S3I-201 were purchased from Merck Millipore (Billerica, MA, USA). The rabbit anti-human STAT3 (79D7) and anti-human phosphorylated STAT3 (D3A7) monoclonal antibodies were purchased from Cell Signaling Technology (Danvers, MA, USA). The mouse anti-human Fascin (D-10) monoclonal antibody, human Fascin siRNA, STAT3 siRNA, negative control siRNA (NC siRNA), and STAT3 inhibitor, Stattic, were purchased from Santa Cruz Biotechnology (Santa Cruz, CA, USA). The CCK8 cell proliferation kit was purchased from Dojindo Molecular Technologies (Kumamoto, Japan). The annexin V-FITC apoptosis detection kit was purchased from Sigma-Aldrich (St. Louis, MO, USA). The Transwell chambers (8-μm pore size) were purchased from Costar (Cambridge, MA, USA).

### Animals

Six-week-old athymic nude mice were obtained from SIPPR-BK Experimental Animal Co. (Shanghai, China). The mice were housed in a pathogen-free facility. The experimental protocols were reviewed and approved by the Animal Care and Use Committee of Zhejiang Cancer Hospital.

### Cells and cell culture

The human GC cell lines AGS and MNK-45 were obtained from the American Type Culture Collection (Manassas, VA, USA) and maintained in 1640 culture medium containing 10% fetal bovine serum (FBS) at 37°C with 5% CO_2_.

### Western blot analysis

A total of 20 μg crude proteins extracted from cell lysates was separated by 10% sodium dodecyl sulfate polyacrylamide gel electrophoresis and transferred onto polyvinylidene difluoride membranes (Millipore, Billerica, MA). The membranes were blocked with 5% BSA in Tris-buffered saline plus 0.05% Tween-20 and then incubated with corresponding primary antibodies at 4°C overnight. After washing with Tris-buffered saline plus 0.05% Tween-20, the membranes were incubated with corresponding HRP-conjugated secondary antibodies. Proteins were visualized using SuperSignal West Femto Maximum (Thermo, IL, USA).

### Detection of cell apoptosis

AGS cells (2 × 10^5^/ml) were treated with 1 or 5 μg/ml of anti-Fas monoclonal antibody (anti-Fas) or isotype antibody (ISO) for 24 h, the apoptotic cells were stained with Annexin V-FITC and propidium iodide for 5 min at 4°C in dark and then analyzed by flow cytometry with a FACSCalibur flow cytometer (Becton Dickinson, San Jose, CA, USA).

### Cancer cell migration assay *in vitro*

AGS cells (1 × 10^6^/ml) were treated with 5 μg/ml of anti-Fas or ISO for 2 h at 37°C. Then 2 × 10^5^ AGS tumor cells were transferred into 100 μl of serum-free media and seeded on to the top chamber of Transwell chambers (8-μm pore size). The bottom chamber was filled with 800 μl of 1640 culture medium containing 20% FBS. After a 48-h incubation at 37°C, the cells were fixed with methanol for 20 min, and washed with PBS thrice, 20 min each. The fixed cells were stained with 10 mg/ml of DAPI for 30 min and washed with PBS. The stained cells were examined under a florescence microscope. To determine the effects of STAT3 on the cell migration, 10 μM of Stattic was added to the culture medium. To determine the role of Fascin in the cell migration, before anti-Fas stimulation, a total of 40 nM Fascin siRNA duplexes were transfected into AGS cells (2 × 10^5^/well) using 3 μl of INTERFERin siRNA transfection reagent (Polyplus, NY, CA, USA) on a 24-well plate. The efficiency of Fascin transient silencing was confirmed by Western blotting.

### Cell proliferation assay

AGS cells were treated with 5 μg/ml anti-Fas or ISO for 24, 48, or 72 h, and 20 μl of CCK8 was added to each well and the cells were incubated for an additional 4 h. The absorbance of each well was read at 450 nm. Cell proliferation was calculated by dividing the ODs of the treated cells with the ODs of the control cells.

### Real-time PCR

Total RNA was extracted with the TRIzol reagent and cDNA was synthesized using a PrimeScript RT reagent kit (Takara Bio, Inc. Otsu, Shiga, Japan). The following PCR conditions were used: 1 cycle at 95°C for 30 s and then 40 cycles of 5 s at 95°C and 34 s at 60°C. Real-time PCR was performed on an Applied Biosystems 7500 real-time PCR system (Foster City, CA, USA). The results were normalized against β-actin RNA. The sequences of PCR primers used were as follows: sense, 5’-CCACGAAACTACCTTCAACTCC-3’ and anti-sense, 5’-GTGATCTCCTTCTGCATCCTGT-3’ for β-actin; sense, 5’-GTGAGGGAAGCGGTTTACGA-3’ and anti-sense, 5’- AGATGCCCAGCATGGTTGTT-3’ for Fas; sense, 5’-TGTCTGCCAATCAGGACGAG-3’ and anti-sense, 5’-CACGCCACTCGATGTCAAAG-3’ for Fascin.

### Lung metastasis assay *in vivo*

AGS cells (5 × 10^6^ per mouse) pre-treated with anti-Fas or ISO for 2 h were injected into 6-wk-old nude mice via a tail vein. To determine the effects of STAT3 on tumor metastasis and Fascin expression *in vivo*, AGS tumor cells (5 × 10^6^ per mouse) were intravenously injected into 6-wk-old nude mice and 24 h later, the mice received intravenous injection of S3I-201 (5 mg/kg, every 2 days for a total of 3 times). Three weeks after cell injection, the mice were anaesthetized by inhaling chloral hydrate and sacrificed, and the lungs were removed and the numbers of lung tumor foci were counted under a dissecting microscope.

### Immunohistochemistry

The tumor foci from the lungs were fixed in 10% formalin, dehydrated in ethanol, and embedded in paraffin. Tissue sections were cut at 4 μm, mounted on slides and dried at 60°C for 4 h. Following short proteolytic digestion and a peroxidase block of the tissue slides using 2.5% hydrogen peroxide in methanol for 30 min at room temperature, the slides were incubated with the anti-Fascin antibody overnight at 4°C. After washing, the slides were incubated with peroxidase-labeled polymer and substrate chromogen. Finally, the specimens were incubated in phosphate buffered saline containing diaminobenzidine for 5 min. An Olympus microscope was employed to visualize the staining of the tumor tissues.

### Statistical analysis

Results were compared using one-way ANOVA. The Spearman rank order correlation test was used to examine correlations between the levels of Fas and Fascin mRNA in tumor tissues from GC patients. A p value of <0.05 was considered to be statistically significant.

## Results

### Activation of Fas signaling promotes the migration of AGS cells

**Fig 1:**
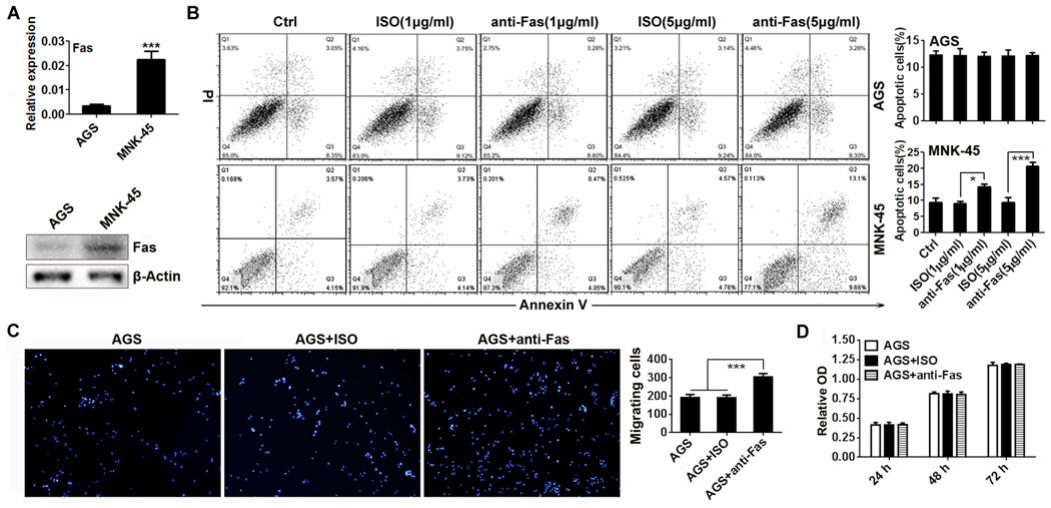
Activation of Fas signaling promotes the migration of GC cells. (A) The Fas expression in AGS and MNK-45 cells were detected by real-time PCR (upper) and Western blot (down). (B) Susceptibility of AGS and MNK-45 cells to Fas-induced apoptosis was measured by staining with Annexin V and PI after both cells were stimulated with anti-Fas or ISO at the indicated concentrations for 24 h (left) and the apoptotic cells were statistically analyzed (right) (n = 3). (C) After stimulated with 5 μg/ml anti-Fas or ISO for 2 h, the AGS cells were collected and seeded into the top chamber. Forty-eight hours later, the number of cells on the bottom of the Transwell filter was imaged (left) and quantified (right) (n = 5). Magnification: 200×. (D) The proliferation of AGS cells was measured by CCK8 assay after stimulation with 5 μg/ml of anti-Fas or ISO in the indicated timepoint. Data are representative of three independent experiments. (*p <0.05, ***p <0.001)

First, we confirmed the expression of Fas receptor in AGS and MNK-45 cells by real-time PCR and Western blotting analyses and found MNK-45 cells expressed higher Fas receptor than AGS cells did (Fig 1A). Both of the cell lines also showed a high level of FasL expression (data not shown). We next examined whether the ligation of Fas receptor could induce apoptosis in AGS and MNK-45 cells. After stimulation with anti-Fas or ISO at a concentration of 1 μg/ml or 5 μg/ml, it was MNK-45 but not AGS cells showed concentration-dependent enhancement of apoptosis (Fig 1B). Therefore, we investigated whether the activation of Fas signaling could cause other effects on AGS cells instead of apoptosis induction in the following experiments. The AGS cell migration assay was performed *in vitro* and we found the migration of AGS cells was significantly increased after stimulation with 5 μg/ml of anti-Fas (Fig 1C). To exclude the possibility that increased migration of AGS cells was caused by their elevated proliferation after anti-Fas stimulation, we examined the proliferation of ACS cells and found no evident difference between cells treated with or without anti-Fas (Fig 1D), suggesting that the increase in cell migration was not a result of the proliferative properties after anti-Fas stimulation. Taken together, these results indicate that Fas signaling can increase the motility of GC cells *in vitro*.

### Activation of Fas signaling upregulated Fascin expression in AGS cells through activation of STAT3

**Fig 2:**
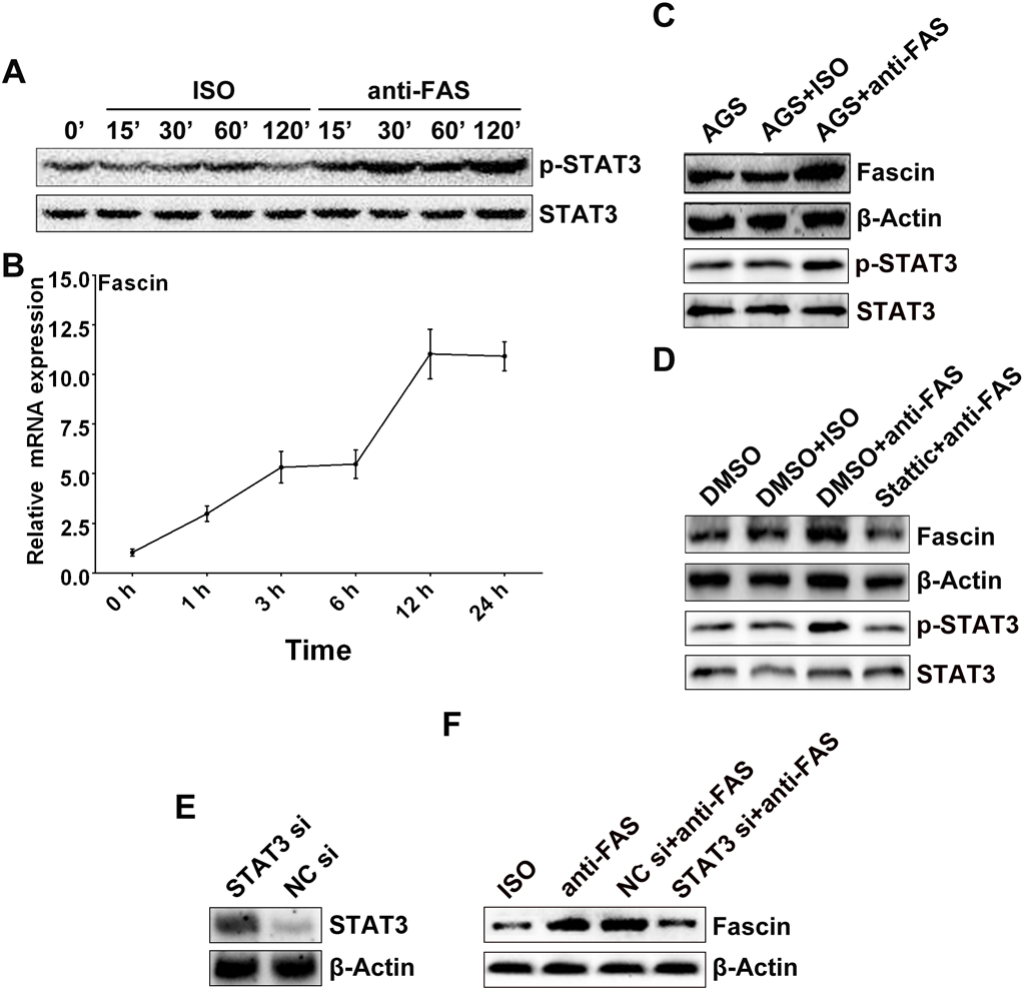
Activation of Fas signaling upregulated Fascin expression in AGS cells through activation of STAT3. The AGS cells were stimulated with 5 μg/ml of anti-Fas in the indicated times. (A) The phosphorylated STAT3 was detected by Western blot. (B) The expression of Fascin mRNA was assayed by real-time PCR. (C) After stimulation with 5 μg/ml anti-Fas for 24 h, the protein level of phosphorylated STAT3 and Fascin in AGS cells was detected by Western blot. (D) The AGS cells were pre-treated with 10 μM of Stattic for 2 h and followed by 5 μg/ml of anti-Fas stimulation for 24 h; the protein level of phosphorylated STAT3 and Fascin was detected by Western blot. After transfection with STAT3 siRNA or NC siRNA for 36 h, (E) the STAT3 expression in the AGS cells was detected by Western blot; (F) the AGS cells were then stimulated with 5 μg/ml of anti-Fas for 2 h, and the Fascin expression in the cells was detected by Western blot. Data are representative of three independent experiments.

It has been reported that Fas signaling is involved in the activation of STAT3 [18–20]. In the present study, we examined whether activation of Fas signaling would cause STAT3 activation in AGS cells. As shown in Fig 2A, after stimulation with anti-Fas for a short period, the phosphorylated STAT3 was significantly increased in AGS cells. It has also been documented that Fascin can increase the motility of tumor cells and is a direct target gene of STAT3 [17]. Thus, we detected the expression of Fascin in AGS cells after stimulation with anti-Fas. As shown in Fig 2B, the mRNA levels of Fascin were increased in a time-dependent manner and culminated after 12 h of anti-Fas stimulation in AGS cells. To further confirm the upregulation of Fascin, we detected the protein level of Fascin and found increasing Fascin protein levels in anti-Fas but not ISO treated AGS cells (Fig 2C). To demonstrate the relationship between STAT3 and Fascin in AGS cells after Fas signaling activation, we treated AGS cells with Stattic, a specific inhibitor of STAT3, before anti-Fas stimulation and found that the anti-Fas-induced increase in Fascin protein was totally abrogated in the presence of Stattic (Fig 2D). We could also detect the activation of STAT3 in AGS cells after anti-Fas stimulation for 24 h, but the activated extent was slightly lower than that received 2h of anti-Fas stimulation (Fig 2C and 2D). To further confirm that STAT3 was involved in the regulation of Fascin expression after anti-Fas stimulation, we knocked down the STAT3 expression by using STAT3 specific siRNA before anti-Fas stimulation and detected the STAT3-knockdown efficacy by Western blotting analyses. As shown in Fig 2E, STAT3 siRNA but not NC siRNA transfection markedly inhibited the STAT3 expression in AGS cells. Expectedly, the Fascin protein was not induced in STAT3-knockdown AGS cells after anti-Fascin stimulation (Fig 2F). These results suggest that activated STAT3 is required for the upregulation of Fascin expression caused by Fas signaling activation in AGS cells.

### Fas signaling-promoted cell migration depends on STAT3/Fascin pathway

**Fig 3:**
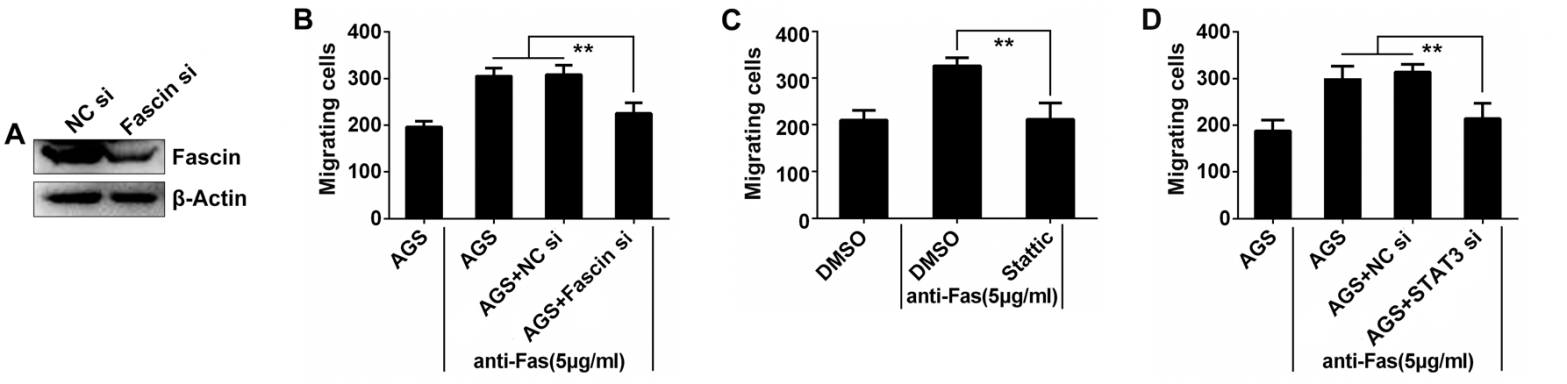
Fas signaling promoted AGS cell migration dependent on STAT3/Fascin pathway. (A) AGS cells were transfected with Fascin siRNA or NC siRNA for 36 h, and Fascin expression in the cells was detected by Western blot. After (B) inhibition of Fascin expression by siRNA; or (C) treated with 10 μM Stattic for 2 h; or (D) inhibition of STAT3 expression by siRNA, and stimulated with 5 μg/ml of anti-Fas for 2 h, the number of AGS cells which migrated to the bottom of the Transwell filter was quantified (n = 5). Data are representative of three independent experiments. (**p <0.01)

It has been shown that Fascin mediates tumor cell migration [17]. In the present study, we examined whether knockdown of Fascin would inhibit Fas signaling-induced cell migration *in vitro*. We found that a decrease in Fascin protein could be detected in AGS cells transfected with Fascin siRNA but not the NC siRNA (Fig 3A). Then, we performed tumor cell migration assays to detect the motility of Fascin-knockdown AGS cells after anti-Fas stimulation. As shown in Fig 3B, when anti-Fas stimulation markedly promoted the migration of AGS cells, this effect was obviously inhibited in cells treated with Fascin siRNA, but not NC siRNA. Since STAT3 is required for the Fas signaling-induced Fascin expression in AGS cells, we next analyzed the effects of STAT3 on the Fas signaling-mediated cell migration in AGS cells. As shown in Fig 3C, after treated with Stattic, Fas signaling-enhanced cell migration was completely abolished. Consistent with this result, STAT3 siRNA also significantly inhibited Fas signaling-enhanced AGS cell migration (Fig 3D). These results indicate that Fas signaling promoted cell migration and it depends on the activation of STAT3/Fascin pathway.

### Fas signaling promotes cell metastasis in vivo through activating STAT3/Fascin pathway

**Fig 4:**
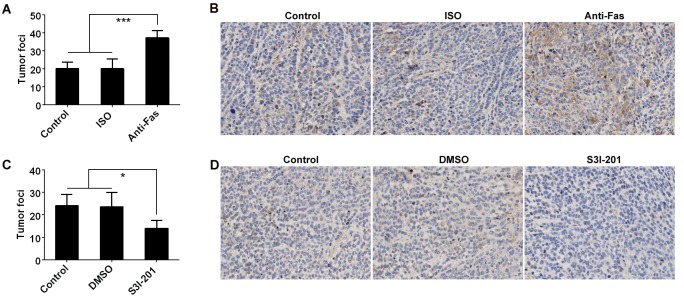
Fas signaling promotes AGS cell metastasis *in vivo* through STAT3/Fascin pathway. 2 × 10^6^ AGS tumor cells pre-stimulated with anti-Fas or ISO for 2 h were intravenously injected nude mice. (A) The number of lung tumor foci was counted (n = 5). (B) The expression of Fascin in tumor tissues from lung was detected by immunohistochemistry. 2 × 10^6^ AGS tumor cells were intravenously injected into nude mice and 24 h later, the mice received intravenous injection of S3I-201 at 5 mg/kg every 2 days for total 3 times. (C) The number of lung tumor foci was counted (n = 5). (D) The expression of Fascin in tumor tissues from lung was detected by immunohistochemistry (magnification: ×100). Data are representative of two independent experiments. (*p <0.05, ***p <0.001)

To further demonstrate the relationship between Fas signaling and GC metastasis, we transferred anti-Fas-stimulated AGS cells intravenously into nude mice and detected the tumor foci in the lungs. We found an increase in tumor foci in the lungs of mice transferred with cells pre-treated with anti-Fas but not ISO (Fig 4A). To clarify the relationship between Fascin and Fas signaling-mediated tumor metastasis, we performed immunohistochemistry detection of Fascin in tumor tissues. We observed a higher Fascin expression in tumor tissues from mice receiving anti-Fas-stimulated AGS cells than that from mice receiving ISO or un-stimulated AGS cells (Fig 4B). Since STAT3 is required for the anti-Fas-induced upregulation of Fascin and increased motility in AGS cells, we investigated the effects of STAT3 on AGS cell metastasis *in vivo*. After treatment with S3I-201, a chemical probe inhibitor of STAT3 activity [21], the tumor foci in the lungs significantly decreased (Fig 4C). In addition, Fascin expression in tumor tissues from S3I-201 treated mice was also inhibited (Fig 4D), suggesting the control of Fascin by STAT3 *in vivo*. Taken together, these results indicate that Fas signaling can promote cell metastasis *in vivo*, dependent on the activation of STAT3/Fascin pathway.

### Fascin expression is correlated with Fas expression in the tumor tissues from patients with GC

**Fig 5:**
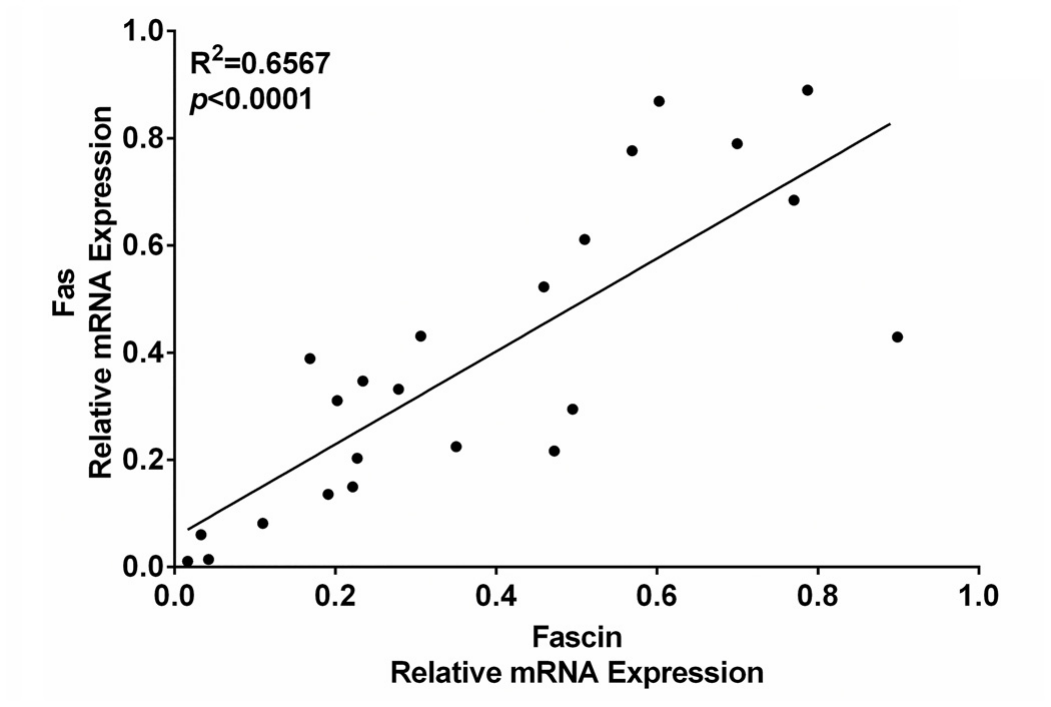
Correlation of the mRNA levels of Fas and Fascin in tumor tissues from GC patients. Fas and Fascin mRNA expression was measured by real-time PCR and normalized to β-actin mRNA (n = 23). Positive correlation was obtained by Spearman correlation analysis.

Since Fas signaling promotes Fascin expression, we determined whether there was a correlation between Fas and Fascin expression in the tumor tissues from GC patients. We analyzed the mRNA levels of Fas and Fascin in the tumor tissues from GC patients. As shown in Fig 5, the mRNA levels of Fas and Fascin showed a positive correlation. This result provides evidence for the notion that Fas signaling promotes Fascin expression in GC.

## Discussion

Generally, following trimerization of Fas after ligation with FasL, apoptosis is initiated. Fas cluster recruits the FADD adapter protein and forms the death-inducing signaling complex, causing the activation of caspase-8. Caspase-8, in turn, activates the downstream caspases, such as caspase-3, culminating in apoptosis [4]. In addition to induce cell death, Fas also transmits proliferation and activation signals in tumor cells [22]. It has been reported that Fas mediating astric mucosal cell proliferation is ERK dependent [23], but activation of ERK signaling pathway cannot induce the proliferation of B16 murine melanoma cells [24]. Therefore, Fas can induce the proliferation of some but not all tumor cells and the relevant mechanism is still largely unknown. Herein, we found Fas was invalid in inducing AGS cell proliferation. Fas signaling has also been demonstrated to induce motility of apoptosis-resistant tumor cells via urokinase plasminogen activator [10]. In the present study, we unraveled a novel mechanism of Fas-mediating tumor cell motility, which depended on the upregulation of Fascin via activation of STAT3.

It is generally believed that to escape apoptosis caused by FasL-positive T cells, tumor cells have developed several ways to resist FasL-induced cell killing effects. Tumor cells have been demonstrated to downregulate or even lose Fas receptor expression [25] or abrogate the intracellular Fas signaling pathway through mutation in Fas [26]. Tumor cells can also upregulate cellular FADD-like IL-1β-converting enzyme-inhibitory protein or phosphorylate caspase-8 to inhibit the activation of caspase-8 and block the down-stream signaling pathway of Fas [27,28]. In the present study, we found the activation of STAT3 in AGS cells after anti-Fas stimulation. The activation of STAT3 has been shown to protect cancer cells from apoptotic stimuli emanating from the Fas receptor [29]. Pre-treatment of STAT3 inhibitor could not initiate AGS cell apoptosis after Fas signaling activation (data not shown), suggesting that the activation of STAT3 is not involved in preventing AGS cells from Fas-induced apoptosis.

It has been reported that Lewis lung carcinoma cells were constitutively resistant to Fas-mediated apoptosis, but the overexpression of Fas on these cells allows Fas-mediated apoptosis after cross-linking with agonist anti-Fas antibody [30], which suggests that there is a qualitative difference in the activated signaling cells receive, which determines their fate after Fas signaling ligation. The level of Fas expression is moderate in AGS cells and stimulated with high dose of anti-Fas (20 μg/ml); we did find that AGS cells showed slightly increased apoptosis (data not shown). This indicates that apoptosis induction and migration promotion signaling may both be activated after Fas receptor ligation, which may be related to the level of anti-Fas used in such experiments. If high levels of Fas-signaling delivery cannot be achieved under physiologic conditions, migration promotion signaling will take over after Fas receptor ligation and cause increased GC metastasis.

To implement remote metastasis, powerful motility is required for the tumor cells. Fascin, as an actin-bundling protein, is important for the maintenance and stability of filamentous actin bundles, and therefore involved in cell motility [31]. In the present study, we revealed that Fas signaling was involved in the upregulation of Fascin expression. IL-6 is reported to upregulate Fascin expression of GC cells [32]. The Fas signaling has also been demonstrated to promote IL-6 secretion in tumor cells [33]. These evidences indicate that Fas signaling probably amplifies the effect of Fascin upregulation through IL-6. We further demonstrated that the Fas and Fascin expressions were closely related in GC patients, strengthening the importance of Fas-Fascin axis in the process of GC metastasis.

Consistent with previous studies, we demonstrated that Fas-induced activation of STAT3 was required for the upregulation of Fascin. In the nude mouse lung metastasis model, the STAT3 inhibitor, S3I-201, could inhibit the Fascin expression in tumor tissues. Murine recombinant FasL was unable to activate STAT3 activation (data not shown), suggesting that activated signaling initiated by FasL on AGS cells themselves is sufficient to mediate STAT3 activation and downstream Fascin upregulation. Besides regulating Fascin expression, STAT3 is also involved in the cell proliferation and survival, oncogenesis, and cancer metastasis in GC [34–36]. An orally bioavailable small-molecule STAT3 inhibitor has been discovered and could be a potential therapeutic agent for human cancer [37]. We speculate that STAT3 inhibitor is a promising agent that can be used in clinical GC therapy in the near future.

In conclusion, we demonstrate that Fas signaling is involved in the GC metastasis through STAT3-dependent upregulation of Fascin. Our results also suggest the Fas/STAT3/Fascin pathway can be a molecular target for GC therapy.
